# A fungal sRNA silences a host plant transcription factor to promote arbuscular mycorrhizal symbiosis

**DOI:** 10.1111/nph.20273

**Published:** 2024-11-18

**Authors:** Alessandro Silvestri, William Conrad Ledford, Valentina Fiorilli, Cristina Votta, Alessia Scerna, Jacopo Tucconi, Antonio Mocchetti, Gianluca Grasso, Raffaella Balestrini, Hailing Jin, Ignacio Rubio‐Somoza, Luisa Lanfranco

**Affiliations:** ^1^ Department of Life Sciences and Systems Biology University of Torino Viale Mattioli 25 10125 Turin Italy; ^2^ Molecular Reprogramming and Evolution (MoRE) Lab Centre for Research in Agricultural Genomics (CRAG) Carrer de la Vall Moronta, 08193 Cerdanyola del Vallès Barcelona Spain; ^3^ Institute of Biosciences and Bioresources, CNR via Amendola 165/A 70126 Bari Italy; ^4^ Department of Microbiology and Plant Pathology, Center for Plant Cell Biology, Institute for Integrative Genome Biology University of California 900 University Avenue Riverside CA 92521 USA

**Keywords:** arbuscular mycorrhizal symbiosis, cross‐kingdom RNA interference, *Medicago truncatula*, *Rhizophagus irregularis*, small RNA

## Abstract

- Cross‐kingdom RNA interference (ckRNAi) is a mechanism of interspecies communication where small RNAs (sRNAs) are transported from one organism to another; these sRNAs silence target genes *in trans* by loading into host AGO proteins. In this work, we investigated the occurrence of ckRNAi in Arbuscular Mycorrhizal Symbiosis (AMS).
- We used an *in silico* prediction analysis to identify a sRNA (*Rir2216*) from the AM fungus *Rhizophagus irregularis* and its putative plant gene target, the *Medicago truncatula MtWRKY69* transcription factor. Heterologous co‐expression assays in *Nicotiana benthamiana*, 5′ RACE reactions and AGO1‐immunoprecipitation assays from mycorrhizal roots were used to characterize the *Rir2216–MtWRKY69* interaction. We further analyzed *MtWRKY69* expression profile and the contribution of constitutive and conditional *MtWRKY69* expression to AMS.
- We show that *Rir2216* is loaded into an AGO1 silencing complex from the host plant *M. truncatula*, leading to cleavage of a host target transcript encoding for the MtWRKY69 transcription factor. *MtWRKY69* is specifically downregulated in arbusculated cells in mycorrhizal roots and increased levels of *MtWRKY69* expression led to a reduced AM colonization level.
- Our results indicate that *MtWRKY69* silencing, mediated by a fungal sRNA, is relevant for AMS; we thus present the first experimental evidence of fungus to plant ckRNAi in AMS.

## Abstract 2

See also the Commentary on this article by Xie & Fan, **246**: 814–817.

## Introduction

RNA interference (RNAi) is a biological process, almost universally present in eukaryotes, which, based on the recognition of target nucleic acids by small RNAs (sRNAs), leads to regulation of gene expression at the transcriptional and/or posttranscriptional level. Small RNAs and RNAi were shown to play a role in different interspecies, and even inter‐kingdom, communication as sRNAs can move from one organism to a distantly related one, leading to the silencing of target genes through the exploitation of RNAi (Cai *et al*., 2018a). This process, known as cross‐kingdom RNAi (ckRNAi), has been described in many pathogenic interactions involving animal and plant systems (Weiberg *et al*., 2013; Buck *et al*., 2014; Zhang *et al*., 2016; Wang *et al*., 2017b; Shahid *et al*., 2018; Cai *et al*., 2018b; Dunker *et al*., 2020; He *et al*., 2021) and in a few plant mutualistic associations (Ren *et al*., 2019; Wong‐Bajracharya *et al*., 2022). *In silico* work has hinted at the possibility of cross‐kingdom RNAi occurring in the Arbuscular Mycorrhizal Symbiosis (AMS) (Silvestri *et al*., 2019), one of the most widespread and ancient symbiotic associations on the planet (Genre *et al*., 2020). Most land plants, including many crops, engage in this symbiosis with soil fungi from the subphylum Glomeromycotina, which provides key benefits to host plants in both natural and agricultural systems (Genre *et al*., 2020). The cellular and metabolic reprogramming of plant cells upon colonization by AM fungi implies a complex network of transcriptional regulation and molecular signaling. Small RNAs are emerging as essential elements within this gene regulatory network (Ledford *et al*., 2023; Zeng *et al*., 2023). There is also indirect evidence that translocation of sRNA occurs in AMS and may be involved in ckRNAi (Qiao *et al*., 2023). In particular, host‐ and virus‐induced gene silencing techniques have been shown to be successful tools for downregulating fungal genes in mycorrhizal roots, pointing to a movement of functional sRNAs from the plant to the AM fungus (Helber *et al*., 2011; Kikuchi *et al*., 2016). Moreover, the observation of extensive membrane rearrangements and the formation of extracellular vesicles at the plant‐AM fungal interface (Ivanov *et al*., 2019; Roth *et al*., 2019) bolsters curiosity since in pathogenic interactions, extracellular vesicles represent a pathway of cross‐kingdom communication and sRNA transfer in ckRNAi (Buck *et al*., 2014; Cai *et al*., 2018a, 2018b; He *et al*., 2023).

In a previous study, we investigated the potential for fungal‐to‐plant sRNA transfer and showed that the model AM fungus *Rhizophagus irregularis* possesses RNAi machinery and produces functional sRNAs, with some predicted to potentially target mRNAs from the host plant *Medicago truncatula* (Silvestri *et al*., 2019). In this work, we set up multiple assays to validate the results of an *in silico* sRNA‐target mRNA prediction analysis and offer experimental evidence that a fungal sRNA guides the silencing of a plant gene through ckRNAi, favoring the establishment of AMS.

## Materials and Methods

### *In silico* target predictions

Target prediction analysis was performed with psRNAtarget (default Schema V2) (Dai *et al*., 2018) using Expectation 2.0 as a threshold. As input, we selected the most abundant sRNA species for each sRNA‐generating loci known from our previous work (Silvestri *et al*., 2019) to be upregulated in intraradical relative to extra‐radical mycelium (589 sRNA in total). *Medicago truncatula* (Gaertn.) transcriptome (version Mt4.0V1) was used as a target. Predicted target transcripts were then ranked based on the expectation scores. Target predictions for all sRNA are listed in Supporting Information Table S1. Target predictions for *Rir2216* are listed in Table S2. To confirm MtWRKY69 as a target of *Rir2216*, we used an additional prediction tool, WMD3, Web MicroRNA Designer (weigelworld.org).

### Sequence conservation analysis

MtWRKY69 orthologues were downloaded from EnsemblPlants (accessed April 2023); 98 protein sequences were obtained. Protein sequences were aligned with mafft v.7.511 (option ‐‐ auto) (Katoh & Standley, 2013), and their phylogenetic relationship was inferred with IQ‐Tree 2.0.7 (options: ‐m TEST ‐bb 1000 ‐alrt 1000) (Minh *et al*., 2020) with the tree rooted on the outlier sequence of *Arabidopsis thaliana* WRKY1. The tree was visualized with the ggtree package (Yu *et al*., 2017) in an R environment. The corresponding CDS sequences were downloaded from EnsemblPant and used for predicting the presence of the *Rir2216* target with psRNATarget (default Schema V2) (Dai *et al*., 2018) using Expectation 2 as a threshold. The CDS sequences from Fabaceae species were aligned with mafft v.7.511 (option ‐‐auto), and two truncated sequences (Vradi0023s00350.1 and Tp57577_TGAC_v2_mRNA20673) were excluded from further analysis. The nucleotide diversity of the aligned CDS was calculated with DnaSP v6 (Rozas *et al*., 2017) using a 21‐mer window‐based method (i.e. Pi for all the possible 21‐mers; from position 1 to the end of the alignment) excluding sites having alignment gaps in the length of the window (i.e. all 21‐mers windows have the same net number of nucleotides).

### Plant material and growth conditions

Seeds of *M. truncatula* A17 Jemalong were scarified on sandpaper and then surface sterilized in 3% sodium hypochlorite. Seeds were then sown onto plant agar and kept in the dark for 16 h at 4°C. Seeds were then brought to a growth chamber at 21°C to germinate. Approximately 1 wk later, seedlings were transferred to sterilized quartz sand media and half of the plants were inoculated with *c*. 2000 spores of *R. irregularis* (DAOM 197198; Agronutrition, Carbonne, France). All the plants were fertilized with a Long Ashton nutrient solution containing 32 μM KH_2_PO_4_ and grown in a climate‐controlled room at 22°C with a photoperiod of 14 h : 10 h, light : dark. Plants were harvested at different time points according to the experiment.

### Generation of *M. truncatula* composite plants

*Medicago truncatula* composite plants were generated following the protocol described by Boisson‐Dernier *et al*. (2001). Seven‐day‐old *M. truncatula* seedlings were punctured in the hypocotyl region using a sterile needle that was dipped in a 48‐h‐old culture of *Agrobacterium rhizogenes* strain Ar1193 carrying the construct of interest inside (a) pK7WG2D vector for expression under CaMV35S promoter or (b) a modified version of pK7WG2D in which the CaMV35S promoter was substituted with 837 bp of the *MtPT4* promoter by *Spe*I digestion and ligation. The seedlings were then placed in L‐S co‐cultivation medium (1.5 g l^−1^ Gamborg B5 Medium, including vitamins ‐Duchefa Biochemie‐; 0.8% plant agar; pH 5.5). The plants were kept in a growth chamber with 14 h : 10 h, 24°C : 20°C, light : dark. After 15 d, the roots that emerged at the infection site were screened for the eGFP protein, the transformation marker gene of the pK7WG2D vector, using a fluorescence stereomicroscope. Nontransformed roots were removed, and plants were transferred to a new L‐S medium containing cefotaxime antibiotic (200 mg l^−1^). The plants were ready to use after 7 d of further incubation in the growth chamber.

### RNA extractions and RT‐PCR assays

Total RNA from roots was extracted using either the Direct‐zol kit (Zymo Research, EuroClone, Pero, Italy), or the Qiagen Plant RNeasy Kit according to the manufacturer's instructions (Qiagen, Milan, Italy). RNA samples were treated with TURBO™ DNase (Thermo Fisher Scientific, Waltham, MA, USA) and checked for DNA contamination through PCR analysis. Single‐strand cDNA was synthesized from 1 μg of total RNA using SuperScript II (Invitrogen) according to the instructions in the user manual. Quantitative reverse transcription polymerase chain reaction was performed using a Rotor‐Gene Q 5plex HRM Platform (Qiagen). All reactions were performed on at least three biological and three technical replicates. Baseline range and take‐off values were automatically calculated using Rotor‐Gene Q 5plex software. Transcript levels were normalized to *M. truncatula* Translation Elongation Factor (TEF). Only take‐off values leading to a mean with a SD below 0.5 were considered. Stem‐loop reverse transcription polymerase chain reaction assays were performed following Varkonyi‐Gasic *et al*. (2007). Briefly, RNA was annealed to stem‐loop RT primers (Table S3) at 65°C for 5 min and then cooled on ice for 1 min. First‐strand cDNA was generated using SuperScript III in a thermocycler (16°C 30 min, 30°C 30 s, 42°C 20 s, 50°C 1 s, 60 cycles followed by 85°C 10 min). The resulting cDNA was then diluted 1 : 10 with water and used as a template for either endpoint PCR or quantitative polymerase chain reaction using primers listed in Table S3.

### Co‐expression assays in *N. benthamiana*

The CDS of *MtWRKY69* was obtained from *M. truncatula* cDNA by PCR using primers listed in Table S3. The sequence for *Rir2216* was cloned into the *M. truncatula* mir159b or the *A. thaliana* miR319a backbones by overlapping PCR, according to Devers *et al*. (2013) or WMD3, respectively (oligonucleotides in Table S3). Using Gateway Cloning, sequences were first inserted into an entry vector (pENTR‐TOPO). PCR‐positive colonies were confirmed by Sanger sequencing. The constructs were then inserted into their destination vectors (pEarleyGate 101 for *MtWRKY69* and pEarleyGate 100 for *Rir2216*) using the LR reaction. Again, PCR‐positive colonies were confirmed by Sanger Sequencing. Destination vectors were then isolated and transformed into *Agrobacterium tumefaciens* strain C58C1. Seeds of *N. benthamiana* were germinated on plant agar plates and transferred to soil after a week. The plants were then grown for 2 additional weeks at a short day (8 h : 16 h, light : dark), light intensity 80–100 μmol m^−2^ s^−1^, at 21°C. *Agrobacterium tumefaciens* was inoculated overnight in 5 ml LB media with appropriate antibiotics. 1 ml of the overnight culture was used to inoculate 25 ml of LB and again left to grow overnight. The culture was centrifugated at 5000 ***g*** for 15 min and then homogenized in resuspension solution (10 mM MgCl_2_, 10 mM MES‐K pH 5.6, 100 μM acetosyringone) and left at RT for 4 h. The A_600_ of each culture was then measured and adjusted to 0.4 (1 : 1 sRNA : target molar ratio for the *A. thaliana* backbone and 3 : 1 for the *M. truncatula* backbone, respectively) for the co‐expression. The infiltration was performed using a needleless syringe applied to the underside of the leaves of *N. benthamiana*. Six leaf discs corresponding to the agroinfiltrated area were used for protein extraction. Target protein levels were visualized with a Western Blot using an anti‐GFP (Roche) antibody according to standard protocols.

### Laser microdissection

Root segments were taken from mycorrhizal and nonmycorrhizal plants (60‐d post inoculation) and collected in RNase‐free tubes containing freshly prepared cold Farmer's fixative (absolute ethanol/glacial acetic acid, 3 : 1 v/v). Root segments were then subjected to vacuum at RT for 20 min. The fixative solution was then changed, and the samples were incubated overnight at 4°C. Samples were then dehydrated in a graded ethanol series (70%, 90% in sterilized water and 100% twice) followed by two steps in Neo‐Clear® (Merck, Darmstadt, Germany), each step for 30 min on ice. The Neo‐Clear was then gradually replaced with paraffin, adding *c*. 10 pieces to 20 ml of Neo‐Clear. Samples were kept at room temperature for 2 h and then put into an oven at 58°C until all paraffin had dissolved. The mixture was substituted with pure paraffin (Paraplast Plus; Sigma‐Aldrich, St Louis, MO, USA), previously melted at 58°C, and then incubated overnight at 58°C with the lid off to aid in the evaporation of remaining Neo‐Clear. The next day, the medium was replaced 1–2 times at *c*. 6 h intervals and paraffin‐containing root pieces were poured into 60 mm Petri dishes and solidified by cooling. Sections of 12 μm were prepared using a rotary microtome. The ribbons of the cut sections were placed on sterile ddH_2_O on an RNase‐free PEN foil slide and dried on a 40°C warming plate. The quality of slides was checked using a bright field microscope using standard glass slides before preparing the slides used for microdissection. Slides were then stored at 4°C before use. A Leica LMD 6500 Laser Microdissection system was used to isolate cells from the tissue sections according to Balestrini & Fiorilli (2020). For each biological replicate, *c*. 1500 cortical cells were collected per cell type. Total RNA was extracted using the PicoPure RNA isolation kit (Arcturus Engineering, Montain View, CA, USA) and treated with DNase (Turbo DNA‐free kit). To verify the absence of DNA contamination, the RNA was tested in reverse transcription polymerase chain reaction assays using *M. truncatula TEF* housekeeping gene. RNA was then used for quantitative reverse transcription polymerase chain reaction amplification reactions, performed with a Rotor‐Gene Q 5plex HRM Platform (Qiagen), and carried out in a total volume of 25 μl, containing 2 μl RNA, 12.5 μl 2X SYBR Green RT‐PCR Reaction Mix, 0.5 μl of each primer (10 μM; Table S3) and 0.5 μl of iScript Reverse Transcriptase for One‐Step reverse transcription polymerase chain reaction. Briefly, the samples were incubated at 50°C for 10 min, followed by a PCR program of 95°C for 5 min, 50 cycles of 95°C for 10 s, 60°C for 30 s. A melting curve was recorded at the end of the run to rule out the possibility of nonspecific PCR amplifications. Baseline range and take‐off values were automatically calculated using Rotor‐Gene Q 5plex software. Transcript levels were normalized to *M. truncatula* TEF. Only take‐off values leading to a mean with a SD below 0.5 were considered.

### RNA‐immunoprecipitation assays

The cDNA sequence of *M. truncatula* AGO1 was cloned under the CaMV35S promoter into the plant expression vector pK7WG2D using Gateway cloning. The vector was transformed into *A. rhizogenes* strain Ar.1193. This strain was then used to perform root transformation in order to obtain overexpressing composite plants, as described above. *M. truncatula* Myc‐tagged AGO1 was immunoprecipitated from 30 g of 60‐d‐old mycorrhizal roots following Dunker *et al*. (2021). Briefly, root tissue was ground to a fine powder under liquid nitrogen and proteins were extracted with IP extraction buffer (20 mM Tris–HCl pH 7.5, 300 M NaCl, 5 mM MgCl_2_, 0.5% (v/v) NP‐40, 5 mM DTT, 1 tablet of complete EDTA‐free protease inhibitor cocktail (Roche, 4 693 132 001)/50 ml, 5 μl RiboLock RNase Inhibitor (Thermo Fisher Scientific, EO0381)/50 ml, made up to 50 ml with DEPC‐treated water) at a dilution of 1 ml per mg of starting material. The lysate was divided into three equal‐volume fractions. AGO1 was immunoprecipitated using either an *A. thaliana* anti‐AGO1 antibody (Agrisera, Vannas, Sweden) or an anti‐myc‐tag antibody (Agrisera). As a control, immunoprecipitation was also performed without an antibody. The immunoprecipitation was carried out with Protein A beads at 4°C for 2 h. The beads were then washed with IP washing buffer (20 mM Tris–HCl pH 7.5, 300 M NaCl, 5 mM MgCl_2_, 0.5% (v/v) Triton X‐100, 5 mM DTT, 1 tablet protease inhibitor/50 ml, made up to 50 ml with DEPC‐treated water) and then divided into separate aliquots for use in Western blot and RNA extraction. RNA was then extracted from the immunoprecipitated fraction using Trizol and precipitated in ethanol overnight at −80°C. A stem‐loop quantitative reverse transcription polymerase chain reaction assay was performed to amplify fungal sRNAs. Primers for the quantitative reverse transcription polymerase chain reaction assay are listed in Table S3.

### 5′ RLM RACE

To detect the truncated fragment of *MtWRKY69* after *Rir2216*‐mediated cleavage we used the FirstChoice RLM‐RACE kit (Life Technologies; Thermo Fisher Scientific). This kit specifically amplifies cDNA from capped mRNA by removing free 5′‐phosphates using alkaline phosphatase and then removing the cap structure from full‐length mRNA using tobacco acid pyrophosphatase. In our experiment, we excluded both treatments so that the 5′ RACE adapter would ligate only to cleaved mRNA fragments. The conditions for first‐strand cDNA synthesis and nested PCRs followed the manufacturer's recommendations. For the first round of nested PCR a forward primer corresponding to the 5′ RACE adapter was used in concert with a reverse primer specific to *MtWRKY69*. Using the PCR product from the first PCR as template, a second round of nested PCR was performed with an inner forward primer on the 5′ RACE adapter and an inner gene‐specific reverse primer. PCR fragments of the expected size were separated by electrophoresis, purified, and cloned into pGEM‐T Easy vectors before being transformed into *E. coli* (TOP10). Following colony PCR, fragments were sent for Sanger sequencing. The oligonucleotides used for detecting the truncated fragment of *MtWRKY69* are listed in Table S3.

### MtWRKY69 overexpression lines

The CDS of *MtWRKY69* or *Scarlet* were amplified and cloned into the pK7WG2D plant expression vector using Gateway cloning. Composite plants were generated using *A. rhizogenes*‐mediated transformation, as described above. Plants were inoculated with *R. irregularis* and after 60 d transformed roots were collected for morphological and molecular analyses of mycorrhiza formation. Roots were stained with cotton blue and the level of mycorrhizal colonization was assessed according to Trouvelot *et al*. (1986) using MYCOCALC (http://www2.dijon.inra.fr/mychintec/Mycocalc‐prg/download.html). Total RNA was extracted and analyzed with quantitative reverse transcription polymerase chain reaction assays as described above. Biological replicates correspond to individual plants.

### Statistical analysis

Statistical tests were carried out through Kruskal–Wallis or One‐way Analysis of Variance (One‐way ANOVA). All statistical elaborations were performed using past statistical package v.4 (Hammer *et al*., 2001).

## Results and Discussion

### The fungal sRNA *Rir2216* is predicted to target the *WRKY69* gene of the host plant *M. truncatula*

**Fig. 1:**
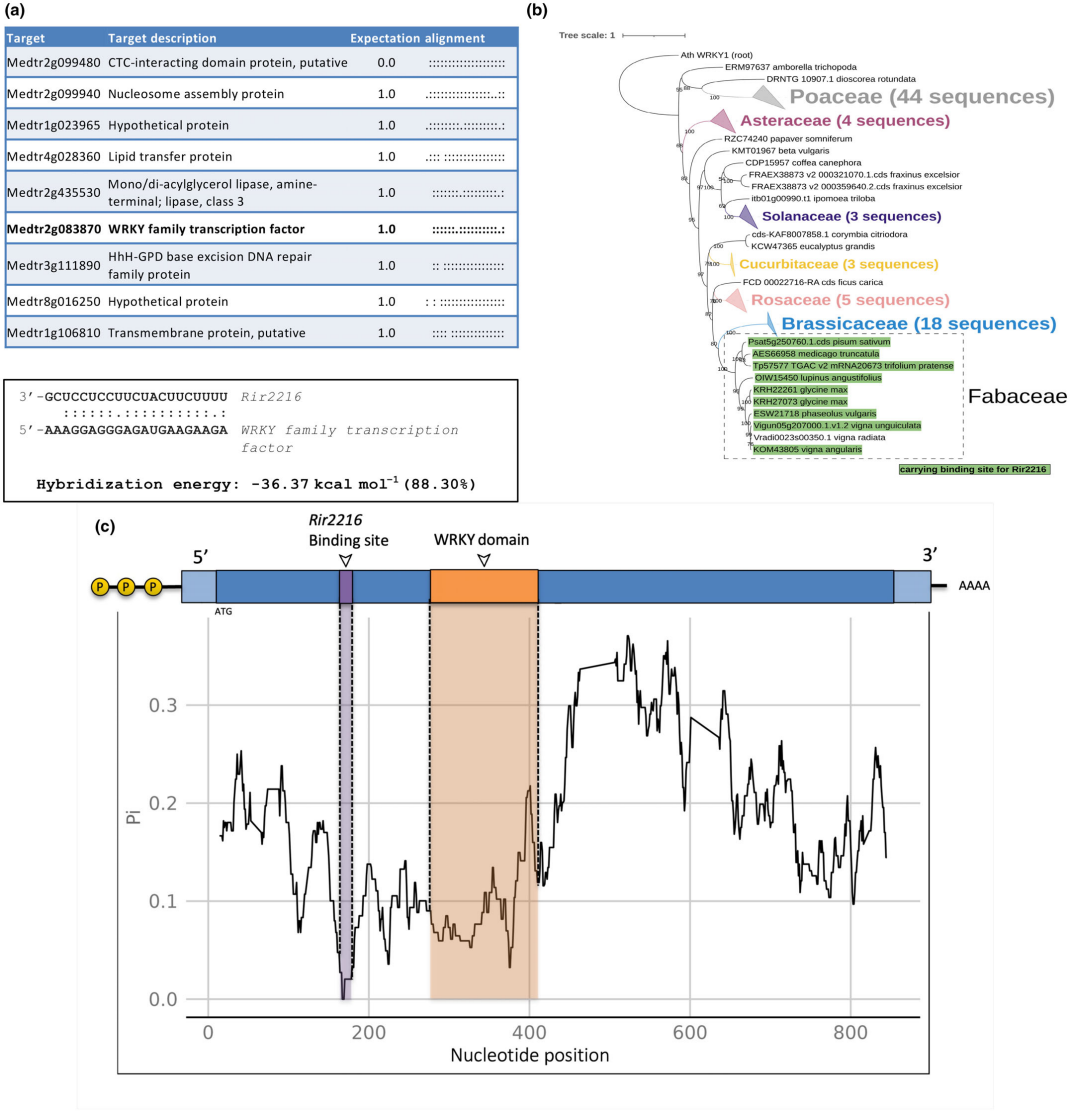
*In silico* analysis of the interaction between *Rir2216* and *MtWRKY69*. (a) (Upper panel) Target prediction results of potential targets of *Rir2216* in the *Medicago truncatula* transcriptome with their associated description, expectation, and alignment. (Lower panel) Alignment of the target site within the *MtWRKY69* sequence with that of *Rir2216* and the associated hybridization energy of the sRNA‐mRNA pair. For the alignment, the following symbols are used: colon (:) denotes standard Watson‐Crick base pairs; dot (.) denotes G‐U wobble pairs. (b) Phylogenetic relationship of MtWRKY69 orthologues. Protein sequences were aligned with mafft v.7.511 (Katoh & Standley, 2013), and their phylogenetic relationship was inferred with IQ‐Tree 2.0.7 (Minh *et al*., 2020) with the tree rooted on the outlier sequence of *Arabidopsis thaliana* WRKY1. Sequences, whose corresponding CDSs contain the *Rir2216* binding site, are indicated in green. (c) Nucleotide diversity (pi) across the CDS alignment of MtWRKY69 orthologous belonging to Fabaceae family, calculated with a 21‐mer sliding‐windows method (i.e. Pi for all the possible 21‐mer across the alignment in steps of 1 nt) excluding sites having alignment gaps. The most conserved 21‐mers in the alignment overlaps with the binding site for *Rir2216*. Notably, the predicted binding site for *Rir2216* has a higher level of conservation than the sequence encoding the WRKY domain.

Using a sequence complementarity approach based on sRNAs from the AM fungus *R. irregularis*, we predicted targets in *M. truncatula* transcriptome. As input, we selected fungal sRNA‐generating loci known to be upregulated in intraradical relative to extra‐radical mycelium (Silvestri *et al*., 2019). We then singled out the most abundant fungal sRNAs from each locus (589 sRNAs in total). Based on target expectation scores, sRNAs from an intergenic locus named ‘cluster_832’ were among the highest ranked (Table S1). After filtering for sRNAs with a length of 21 nucleotides – the length of previously described ckRNAi sRNAs (Cai *et al*., 2018a) – cluster_832 became especially pronounced (Table S1). We named the most abundant sRNA of cluster_832 *Rir2216*. We identified *Rir2216* in other publicly available RNA‐Seq data from *R. irregularis* (Table S4). *Rir2216* presented isoforms ranging from 21 to 24 nucleotides in length as it was observed in other sRNAs involved in cross‐kingdom RNAi (Wang *et al*., 2017a). As revealed by two independent prediction tools, among the highest scoring targets of *Rir2216* was a gene encoding for the *M. truncatula* WRKY transcription factor 69 (*MtWRKY69*; Medtr2g083870, Fig. 1a; Table S2). WRKY transcription factors are known to regulate several aspects of plant biology, including responses to biotic factors (Jiang *et al*., 2017; Chen *et al*., 2019), making *MtWRKY69* a particularly interesting target gene. The theoretical hybridization energy between *Rir2216‐MtWRKY69* is −36.37 kcal mol^−1^ (88.30%) (Fig. 1a), which is within the range of hybridization energies found in endogenous miRNA : target duplexes in plants (Alves‐Junior *et al*., 2009).

We hypothesized that, if *Rir2216*‐dependent regulation of *MtWRKY69* was functional and important for AMS establishment, the binding site of *Rir2216* should be conserved in WRKY69 orthologs from other species able to engage in AMS with *R. irregularis*. To test this, the protein sequences of MtWRKY69 orthologues (98 sequences in total) were retrieved from EnsemblPlant and used to build a phylogenetic tree (Fig. 1b). The MtWRKY69 orthologs clustered into groups based on species phylogeny, including Poaceae, Brassicaceae, and Fabaceae members. Examination of their coding sequences led to the identification of those carrying potential binding sites of *Rir2216* by repeating the target analysis on the orthologues using *Rir2216* as a guide. All but one of the species that clustered to Fabaceae (an AMS‐forming family) presented the predicted binding site for *Rir2216* within the CDS of their orthologous MtWRKY69. The species that clustered to other families did not contain a predicted binding site for *Rir2216*. Among the Fabaceae family orthologs, the nucleotide diversity (Pi index) was calculated using a window‐based method (Pi for all possible 21‐mers; from position 1 to the end of the alignment). The predicted binding sequence of *Rir2216* was found to overlap with the most conserved 21‐mer site in the entire alignment, even more conserved than 21‐mers corresponding to the WRKY domain itself (Figs 1c, S1). Such a degree of conservation supports the biological regulatory relevance of the specific sequence motif. We can speculate that the *Rir2216*–*WRKY* interaction evolved specifically in the Fabaceae family to control mycorrhizal formation in this group of plants. However, as mentioned before, we identified *Rir2216* isoforms in publicly available RNA‐Seq data from *R. irregularis*‐colonized roots of other plant species (*Nicotiana attenuata*, *Solanum lycopersicum*; Table S4); we can hypothesize that in these hosts that belong to the Solanaceae family, *Rir2216* may have other target mRNAs. Such instance has been described for plant miRNAs, where for example miR396 can target GRF, bHLH and/or MADs box transcription factors in different species, being those different targets related to defense responses (Silvestri *et al*., 2024 and references therein). A wider knowledge on the population of sRNA in mycorrhizal roots of different plant–fungus combinations would be instrumental to clarify this issue.

### *MtWRKY69* is a *bona fide* cross‐kingdom RNAi target of *Rir2216*

**Fig. 2:**
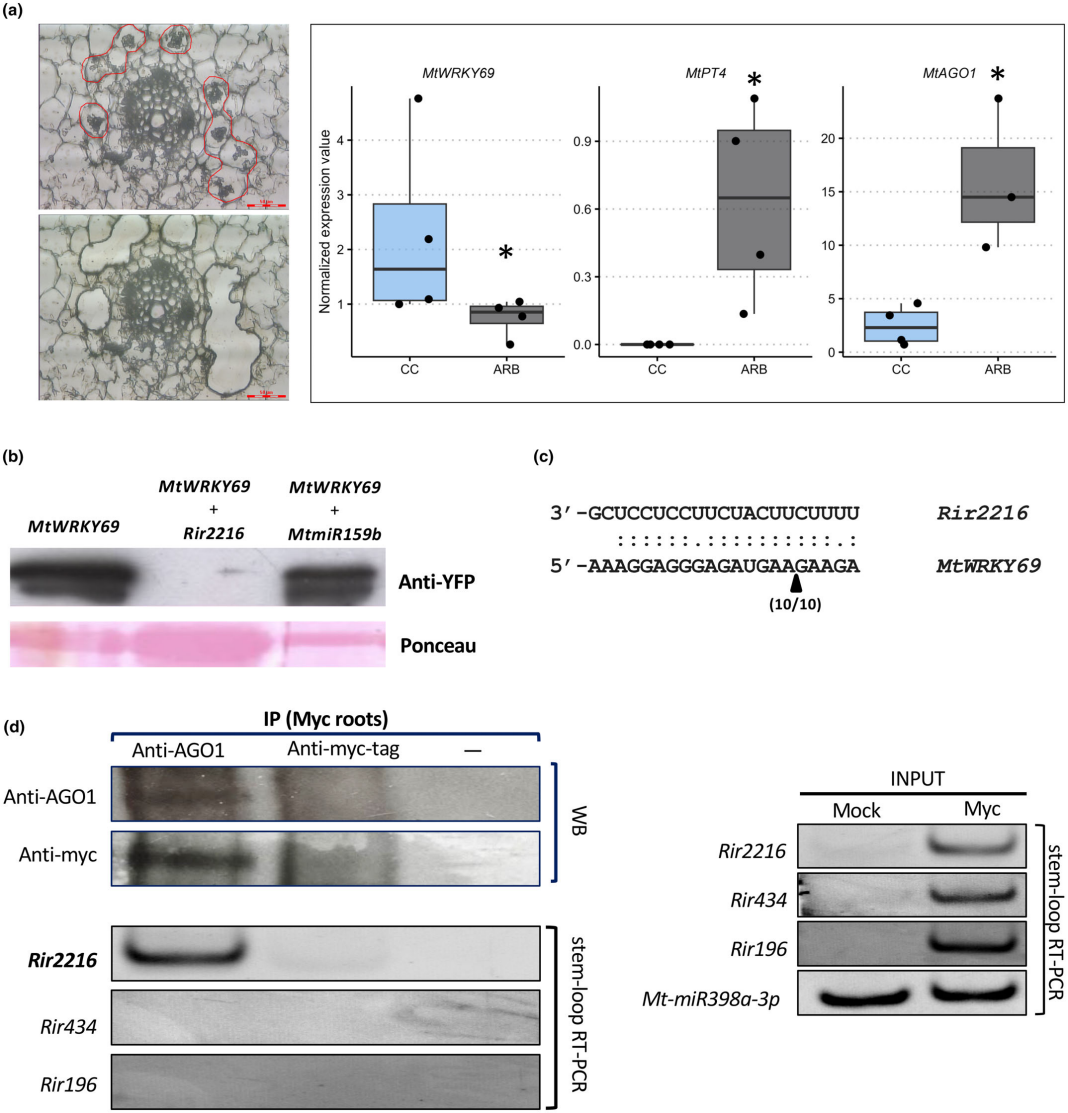
*Rir2216* is a bona fide ckRNAi sRNA targeting MtWRKY69. (a) On the left: transversal section of a mycorrhizal root under the laser microdissector before (upper panel) and after (down) the cut. Arbuscule‐containing cells are indicated by a red line. Bar, 50 mm. On the right: Normalized expression values of *MtWRKY69*, *MtPT4*, and *MtAGO1* transcript abundance in cells collected by laser microdissection: CC‐cortical cells from noncolonized roots; Arb‐cortical cells containing arbuscules. Box plots display the median (horizontal line), the quartiles (boxes) and 1.5 interquartile range (whiskers); each dot corresponds to an independent replicate. Statistical analysis was performed using one‐way analysis of variance (Kruskal–Wallis test; *, *P* < 0.05). (b) Western blot of co‐expression assays using proteins extracted from *Nicotiana benthamiana* leaves expressing WRKY‐YFP alone, in combination with *Rir2216*, or with the plant miR159b. The lower panel corresponds to a Ponceau staining of the gel showing the Rubisco protein. (c) The cleavage site (arrow) and frequencies (indicated by the ratio of the number of clones showing that 5′ end to the total number of sequenced recombinant clones) detected using a 5′ RACE on *Medicago truncatula* mycorrhizal roots. (d) (Upper panel left) Western blot (WB) of anti‐AGO1 (left lane) and anti‐myc‐tag (central lane) and no‐antibody (right lane) immunoprecipitations with anti‐AGO1 and anti‐myc‐tag antibodies. Full images of blots are shown in Supporting Information Fig. S6; (lower panel left) Gel electrophoresis of stem‐loop reverse transcription polymerase chain reaction assays on *Rir2216*, *Rir434*, and *Rir196* using RNA extracted from the immunoprecipitated fractions. (Right) Gel electrophoresis of stem‐loop reverse transcription polymerase chain reaction assays on *Rir2216*, *Rir434*, *Rir196*, and *Mt‐miR398a‐3p* from input RNA from noninoculated (Mock) and mycorrhizal roots (Myc).

If *Rir2216* suppresses the expression of *MtWRKY69* by ckRNAi, a lower abundance of its transcripts in mycorrhizal roots should be observed. Quantitative reverse transcription polymerase chain reaction assays on RNA extracted from whole roots revealed that *MtWRKY69* expression did not change in mycorrhizal samples compared to control roots (Fig. S2). As mycorrhizal roots are a heterogeneous environment consisting of different plant cell types and fungal structures, gene expression profiles associated to specific cell types can be masked by a dilution effect when RNA is analyzed at the whole root level. Laser Microdissection (LMD) technology was therefore exploited to isolate arbuscule‐containing cortical cells (Fig. 2a), which are considered the critical functional structures of the AMS in which fungal and plant cells achieve the most intimate interaction and where the nutrient exchange is thought to occur (Genre *et al*., 2020). The quality and identity of the LMD samples were confirmed by the transcript abundance of *MtPT4*, a phosphate transporter encoding gene specifically expressed in arbusculated cells (Javot *et al*., 2007) (Fig. 2a). *MtWRKY69* was found to be downregulated in arbuscule‐containing cells compared to cortical cells from nonmycorrhizal roots (Fig. 2a). As AGO1 is the nuclease central to posttranscriptional gene silencing and previously described as involved in ckRNAi (Dunker *et al*., 2020) we also monitored the expression of *MtAgo1* in these LMD samples. A significant upregulation of *MtAgo1* in arbusculated cortical cells relative to those from control roots was observed (Fig. 2a), indicating that this component of the plant RNAi machinery is activated in this specific cell type.

To assess whether *Rir2216* is capable of silencing its predicted target *MtWRKY69 in planta*, we set up transient co‐expression assays in *Nicotiana benthamiana* leaves. We inserted *Rir2216* into two miRNA backbones: miR159b from *M. truncatula* (Fig. 2b) and miR319a from *A. thaliana* (Fig. S3). We separately cloned the YFP (yellow fluorescent protein) coding sequence fused to MtWRKY69 at the C‐terminal end (MtWRKY69‐YFP) under the strong and constitutive 35S promoter from the Cauliflower Mosaic Virus (CaMV35S). When MtWRKY69‐YFP was expressed alone, a robust accumulation of the chimeric protein was detected by Western blot (Figs 2b, S3). By contrast, when MtWRKY69‐YFP was co‐expressed with miRNA backbones containing *Rir2216*, a visible decrease in protein accumulation was observed. The decrease in protein accumulation was not observed when MtWRKY69‐YFP was co‐expressed with sRNAs of plant (*MtmiR159b*, Fig. 2b) or fungal (*Rir773*; Fig. S3) origin that lacked predicted target sites in the *MtWRKY69* sequence. These results demonstrate that *Rir2216* is able to silence its target gene, *MtWRKY69*, *in planta* in a sequence‐specific manner.

To confirm that *Rir2216* can target *MtWRKY69* in mycorrhizal roots, we performed a 5′ RLM‐RACE assay to amplify the cleavage fragment of *MtWRKY69* based on predicted AGO‐catalyzed *Rir2216*‐guided endonucleolysis. We first successfully validated the RACE library by corroborating the presence of *MtHB8* cleaved transcripts within the known miR166 binding site (Fig. S4a; Boualem *et al*., 2008). Subsequently, we obtained a unique PCR product showing the expected size when mapping *MtWRKY69* cleaved products (Fig. S4b). Sequencing of the amplified DNA fragment showed that it did indeed belong to *MtWRKY69* and that the 5′‐end was located within the predicted binding site of *Rir2216* (Fig. 2c). Nevertheless, target cleavage mapped 4 nucleotides downstream of a canonical plant miRNA‐guided cleavage site that is found between the nucleotides 10–11 from the 5′‐end of the miRNA. Deviations from the canonical cleavage position are often observed when mapping miRNA‐ and cross‐kingdom sRNA‐mediated cleavage (Llave *et al*., 2002; Jones‐Rhoades & Bartel, 2004; Zhao *et al*., 2012; Tsikou *et al*., 2018; Ren *et al*., 2019; Ji *et al*., 2021). The reason behind the production of noncanonical cleavage fragments around small RNA binding sites is not completely understood. However, it is possible that exonucleases like XRN4 (Souret *et al*., 2004) may trim the cleavage product after AGO‐catalyzed cleavage. Inefficient AGO‐catalyzed target cleavage may also lead to stalling of RISC at the target site. Thus, site‐specific cleavage independent of catalytic activity of AGO, possibly mediated by stalled ribosomes (Arribas‐Hernández *et al*., 2016), could occur following AGO binding.

To verify the possible association of *Rir2216* to components of *M. truncatula* RNAi machinery, we performed RNA immunoprecipitation (RIP) to pull down sRNAs associated with *M. truncatula* AGO1 from mycorrhizal roots, and then followed with stem‐loop reverse transcription polymerase chain reaction to examine specific sRNAs. We focused on AGO1 for three reasons: *Rir2216* contains the hallmarks of AGO1 binding (5′ U and 21 nt in length) (Mi *et al*., 2008), in other biological systems AGO1 is the member of the AGO family principally involved in ckRNAi (Wang *et al*., 2016; Shahid *et al*., 2018; Cai *et al*., 2018a; Cui *et al*., 2019), and we had previously demonstrated upregulation of *MtAGO1* in arbusculated cells (Fig. 2a). We first confirmed the cross‐reactivity of a commercially available *A. thaliana* anti‐AGO1 antibody against *M. truncatula* AGO1 by performing a Western blot using proteins extracted from shoots and mycorrhizal roots. We were able to detect a corresponding band (*c*. 122 kDa) from shoots but not from roots, possibly due to the low concentration of AGO1 in the protein extract (Fig. S5). Therefore, we generated composite plants with roots expressing *MtAGO1* – tagged with a myc epitope at the N‐terminal – under the CaMV35S promoter. Through this approach, the recombinant AGO1 was successfully immunoprecipitated from mycorrhizal roots of composite plants using the *A. thaliana* anti‐AGO1 antibody but not the anti‐myc‐tag antibody, as confirmed by Western blot (Fig. 2d, top left). RNA was extracted from the immunoprecipitated fractions and used for stem‐loop reverse transcription polymerase chain reaction assays. We identified *Rir2216* in the anti‐AGO1‐immunoprecipitated fraction but neither in the anti‐myc‐tag fraction (in which the AGO1 pull‐down was unsuccessful) nor in the control sample without antibody (Fig. 2d, bottom left). As negative controls, two fungal sRNAs, *Rir434* and *Rir196*, were analyzed in parallel. Both sRNAs showed higher expression levels compared to *Rir2216* in the sRNAseq dataset (Silvestri *et al*., 2019) and were detected in the input of RNA immunoprecipitation from mycorrhizal roots (Fig. 2d, right). In addition, *Rir434* possesses similar structural characteristics to known AGO1‐binding sRNAs (Mi *et al*., 2008), while *Rir196* does not. Notably, no amplification was observed in the immunoprecipitated fraction for either *Rir434* or *Rir196*. This suggests that there is selectivity in the transfer of fungal sRNAs to plants and/or their association with host AGO1 proteins during *R. irregularis* root colonization. These findings indicate that *Rir2216* is a functional sRNA involved in ckRNAi.

### Increased levels of *MtWRKY69* expression reduce AM fungal colonization of host roots

**Fig. 3:**
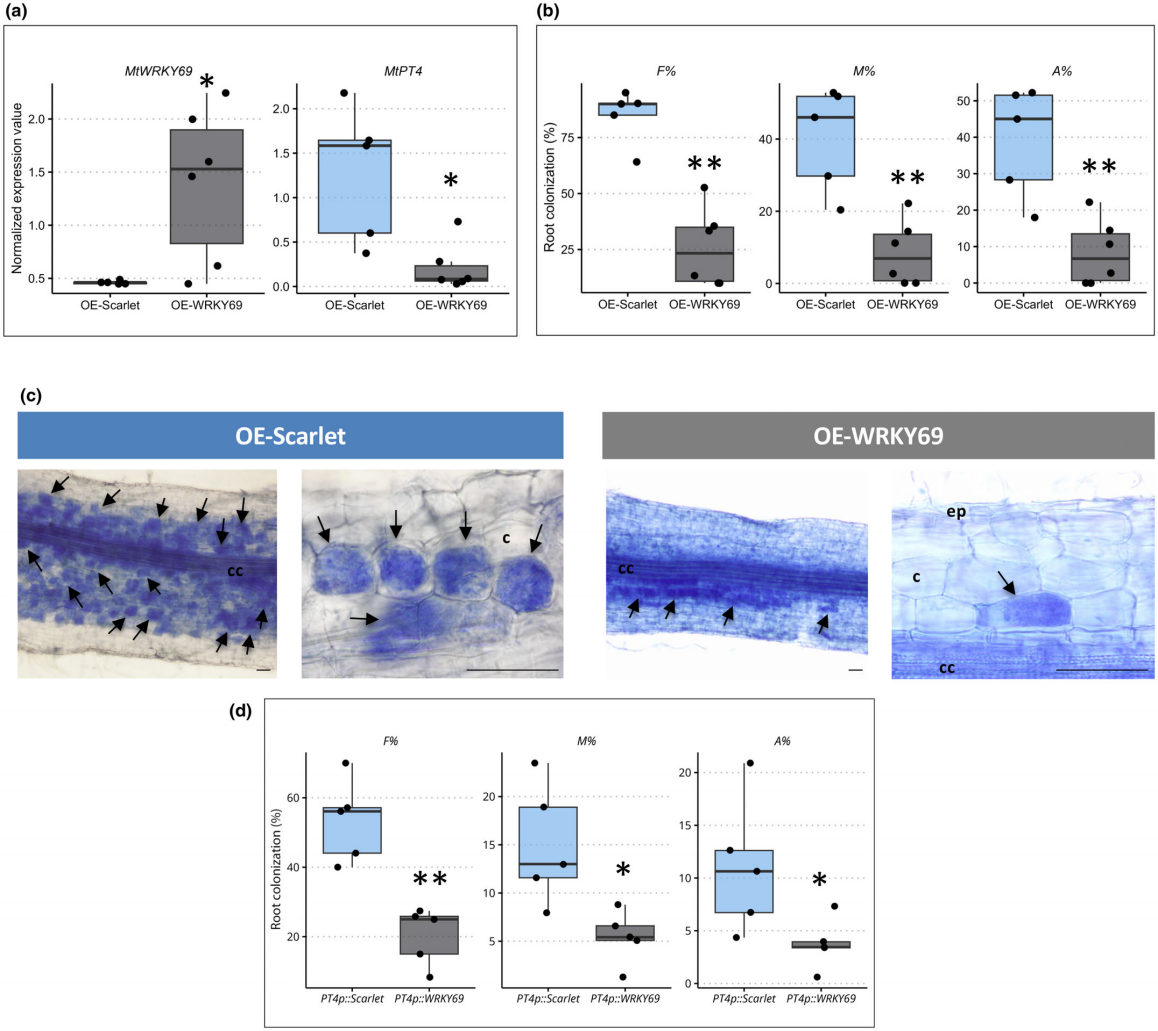
MtWRKY69 transcription factor modulates fungal colonization. (a) Normalized expression value of *MtWRKY69* and the AM‐responsive gene *MtPT4* in plants expressing *Scarlet* or *MtWRKY69* under the CaMV35S promoter (OE = overexpressing). (b) Frequency of mycorrhizal colonization (F%), intensity of colonization (M%) and arbuscules abundance (A%) in composite plants expressing *Scarlet* or *MtWRKY69* under the CaMV35S promoter at 60 d post inoculation (dpi). (c) Representative images of *R. irregularis*‐colonized roots from composite plants overexpressing *Scarlet* or *MtWRKY69*. Arrows indicate arbuscule‐containing cells. cc, central cylinder; c, cortical cells; ep, epidermal cells. Bars correspond to 50 μm. (d) Frequency of mycorrhizal colonization (F%), intensity of colonization (M%) and arbuscules abundance (A%) in composite plants expressing *Scarlet* or *MtWRKY69* under the *MtPT4* promoter at 60 dpi. In a, b and d, box plots display the median (horizontal line), the quartiles (boxes) and 1.5 interquartile range (whiskers); each dot corresponds to an independent replicate. Statistical analysis was performed using one‐way analysis of variance (ANOVA; *, *P* < 0.05; **, *P* < 0.01).

To establish the biological significance of *Rir2216*‐mediated regulation of its target gene, we overexpressed *MtWRKY69* and tested how plants responded to fungal colonization. To that end, we generated composite plants expressing *MtWRKY69* or, as a control, the fluorescent protein Scarlet, under the CaMV35S promoter, and inoculated them with *R. irregularis*. By quantitative reverse transcription polymerase chain reaction, we first confirmed the upregulation of *MtWRKY69* and then observed that *MtWRKY69* overexpression significantly reduced the level of AM colonization as indicated by transcript abundance of the *M. truncatula* AM marker gene *MtPT4* compared to *Scarlet* expressing plants (Fig. 3a). Morphological analyses of mycorrhizal formation confirmed the molecular data: a lower percentage of frequency, intensity and arbuscule abundance was observed in *MtWRKY69*‐overexpressing plants compared to control plants (Fig. 3b). No alteration in arbuscule morphology was observed (Fig. 3c). As the overexpression of the gene driven by the CaMV35S promoter could lead to pleiotropic effects, which, in turn, might affect AM symbiosis development, we also analyzed the mycorrhizal phenotype of composite plants expressing *MtWRKY69* under the arbuscule‐specific promoter of *MtPT4*, along with *Scarlet* as control. *MtPT4* promoter conditionally increased the expression of *MtWRKY69* or *Scarlet* solely upon AMS, resulting again in a reduced mycorrhizal formation level only when MtWRKY69 was induced (Fig. 3d).

On the whole, these results indicate that regulation of *MtWRKY69* expression levels plays a role in controlling the extent of fungal colonization in mycorrhizal roots.

### Conclusions

In summary, our work describes fungus to plant ckRNAi in the AMS for the first time. Starting with *in silico* target predictions, we identified a plausible ckRNAi interaction between the fungal sRNA *Rir2216* and the plant transcript *MtWRKY69*. We propose that *R. irregularis* exports *Rir2216* to cortical cells which establish intimate contact with the fungus, and, by hijacking the AGO1‐equipped plant RNA silencing machinery, *Rir2216* downregulates the *MtWRKY69* encoding gene at the posttranscriptional level. As many WRKY transcription factors are involved in the response to pathogens (Jiang *et al*., 2017; Chen *et al*., 2019), we speculate that the *Rir2216*‐mediated silencing of *MtWRKY69*, particularly in cortical cells that house arbuscules, could contribute to local suppression of the plant immune response, which would favor successful colonization. This comports with the previously described mechanism of action of the SP7 protein effector in *R. irregularis* (Kloppholz *et al*., 2011). Further investigations on the function and regulation of *MtWRKY69* in more detail are needed to elucidate its specific regulatory role in AMS. In addition, it so far remains unknown how *R. irregularis* exports *Rir2216*, and possibly other sRNAs, into plant cells; indeed, whether ckRNAi is mediated by extracellular vesicles, ribonucleotide binding proteins or passive diffusion remains to be deciphered (Zand Karimi *et al*., 2022; He *et al*., 2023). Finally, whether ckRNAi in the AMS is a bidirectional phenomenon, as has been previously reported in other interactions (Weiberg *et al*., 2013; Wang *et al*., 2016; Zhang *et al*., 2016; Cai *et al*., 2018a; He *et al*., 2023), is still unknown. Taken together, our findings describe a new layer of plant–fungus communication in the AMS and are a stimulus for further research into the molecular mechanisms underlying one of the most important symbioses on the planet.

## Competing interests

None declared.

## Author contributions

A. Silvestri, WCL and LL conceived and designed the study. A. Silvestri, WCL, VF, CV, RB, A. Scerna, JT, AM and GG performed the experiments and analyzed the data. VF, RB, HJ and IR‐S contributed to experimental design, with valuable intellectual input. A. Silvestri, WCL IR‐S and LL wrote the manuscript. LL supervised the project. A. Silvestri and WCL contributed equally to this work. All authors have read and approved the final version of the manuscript.

## Supporting information


**Fig. S1** Sequence conservation analysis.
**Fig. S2** Time‐course expression analysis.
**Fig. S3** Co‐expression assay using fungal sRNAs in *Arabidopsis thaliana* amiRNA backbone miR319A.
**Fig. S4** 5′ RACE assay.
**Fig. S5** Western blot using the anti‐AGO1 antibody on proteins extracted from *Medicago truncatula* mycorrhizal samples.
**Fig. S6** Entire blots from Fig. 2(d) with anti‐AGO1 (upper) and anti‐myc (lower) antibodies.

**Table S1** Target prediction of fungal sRNAs upregulated in the intraradical mycelium.
**Table S2** Predicted targets of *Rir2216*.
**Table S3** List of oligonucleotides.
**Table S4** *Rir2216* isoform abundance.
Please note: Wiley is not responsible for the content or functionality of any Supporting Information supplied by the authors. Any queries (other than missing material) should be directed to the *New Phytologist* Central Office.

## Data Availability

The data that support the findings of this study are available in the Supporting Information.
