# Supplementary material for: A fungal sRNA silences a host plant transcription factor to promote arbuscular mycorrhizal symbiosis
Source: New Phytol. 2024 Nov 18;246(3):924–35. doi: 10.1111/nph.20273 (PMC11982788; doi:10.1111/nph.20273)
Supplement: Supplementary file 1 — Fig. S1 Sequence conservation analysis. Fig. S2 Time‐course expression analysis. Fig. S3 Co‐expression assay using fungal sRNAs in Arabidopsis thaliana amiRNA backbone miR319A. Fig. S4 5′ RACE assay. Fig. S5 Western blot using the anti‐AGO1 antibody on proteins extracted from Medicago truncatula mycorrhizal samples. Fig. S6 Entire blots from Fig. 2(d) with anti‐AGO1 (upper) and anti‐myc (lower) antibodies. [file NPH-246-924-s001.pdf]

New Phytologist Supporting Information

Article title: A fungal sRNA silences a host plant transcription factor to promote arbuscular mycorrhizal symbiosis

Authors: Alessandro Silvestri, William Conrad Ledford, Valentina Fiorilli, Cristina Votta, Alessia Scerna, Jacopo Tucconi, Antonio Mocchetti, Gianluca Grasso, Raffaella Balestrini, Hailing Jin, Ignacio Rubio-Somoza, Luisa Lanfranco

Article acceptance date: 24 October 2024

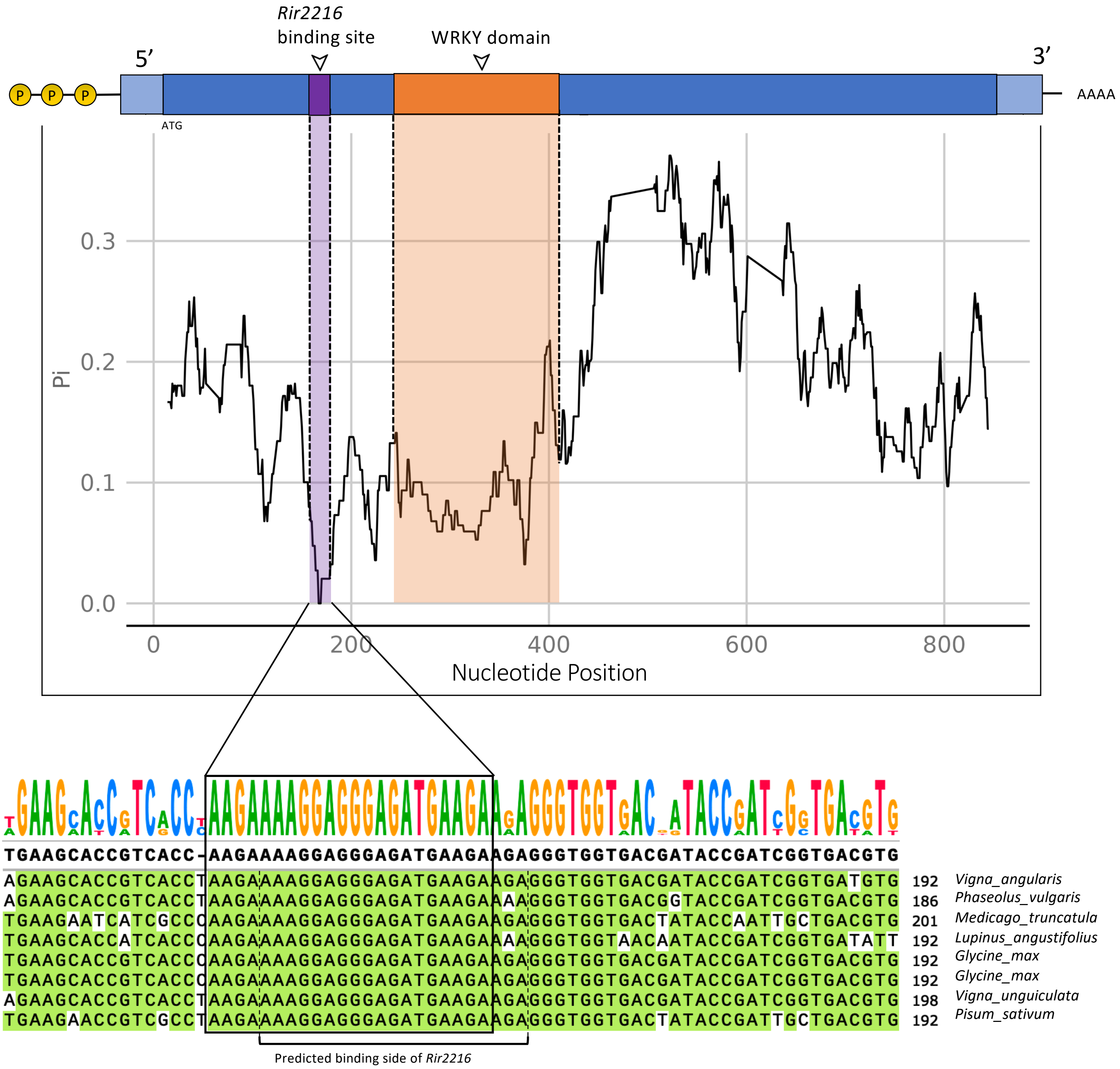

Fig. S1. Sequence conservation analysis. Detail of the alignment obtained using CDS sequences from Fabaceae species with MAFFT v7.511 (Katoh & Standley, 2013) over the predicted binding site of *Rir2216*.

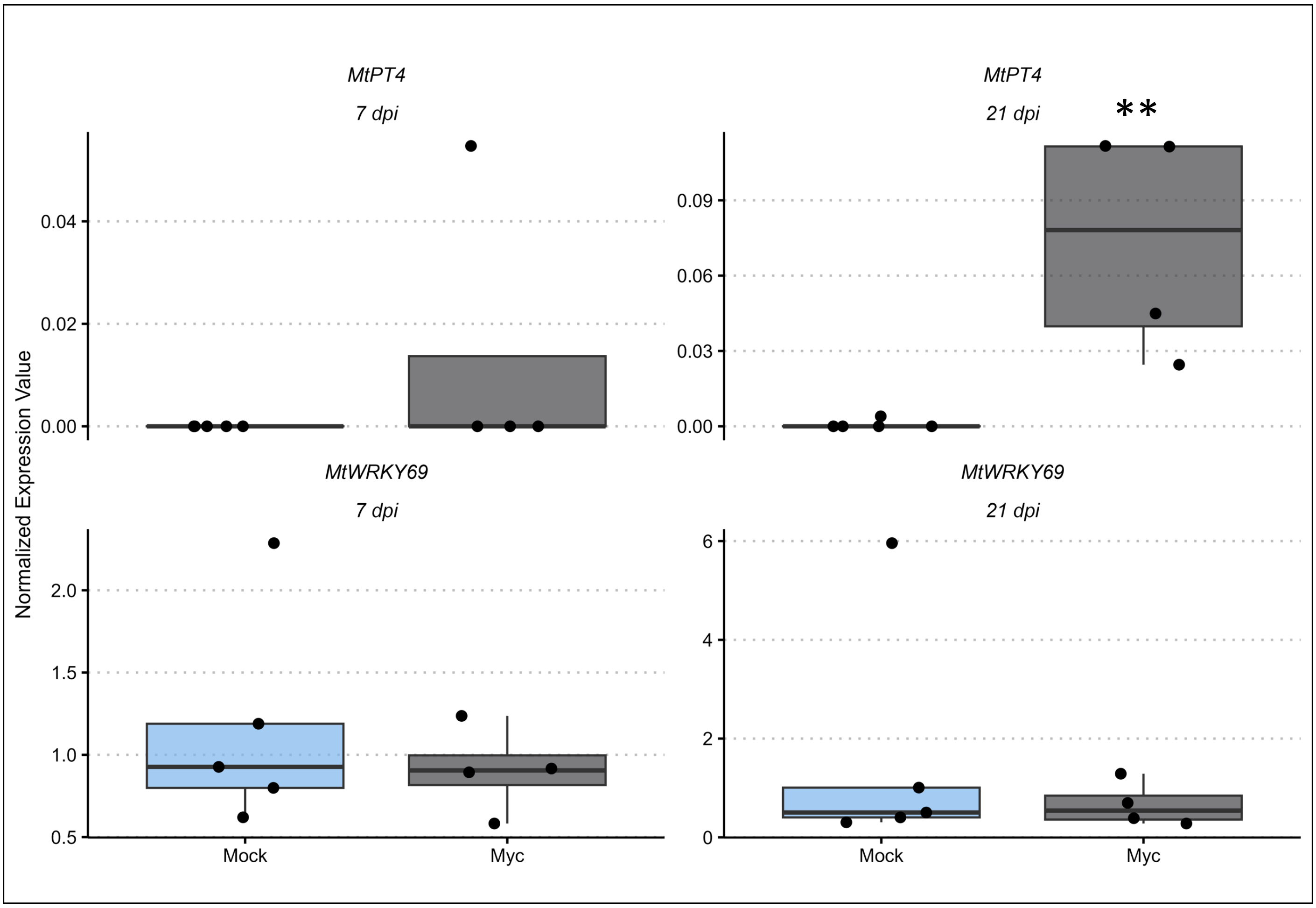

Fig. S2. Time-course expression analysis. Normalized expression values of *MtWRKY69* and the AM-responsive gene *MtPT4* on mock-treated (Mock) and mycorrhizal (Myc) roots at early (7 dpi -day post inoculation-) and late (21 dpi) stages of mycorrhization. Each dot corresponds to an independent replicate. Statistical analysis was performed using one-way analysis of variance (ANOVA; **P<0.01).

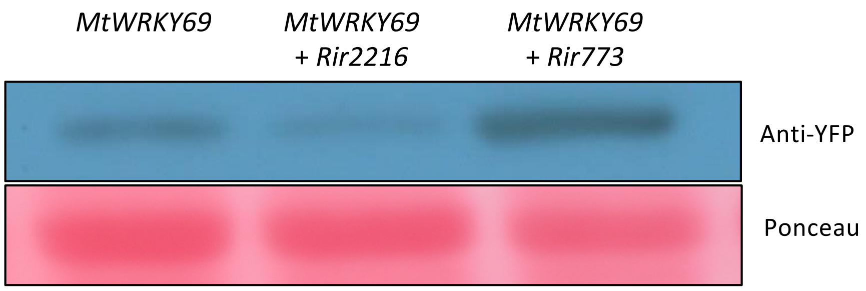

Fig. S3. Co-expression assay using fungal sRNAs in *A. thaliana* amiRNA backbone miR319A. Western blots of co-expression assays using proteins extracted from *N. benthamiana* leaves expressing WRKY-YFP alone, in combination with *Rir2216,* or with the fungal sRNA *Rir773,* as a negative control, from the *A. thaliana* amiRNA backbone miR319A. The lower panel corresponds to a Ponceau staining of the gel showing the Rubisco protein.

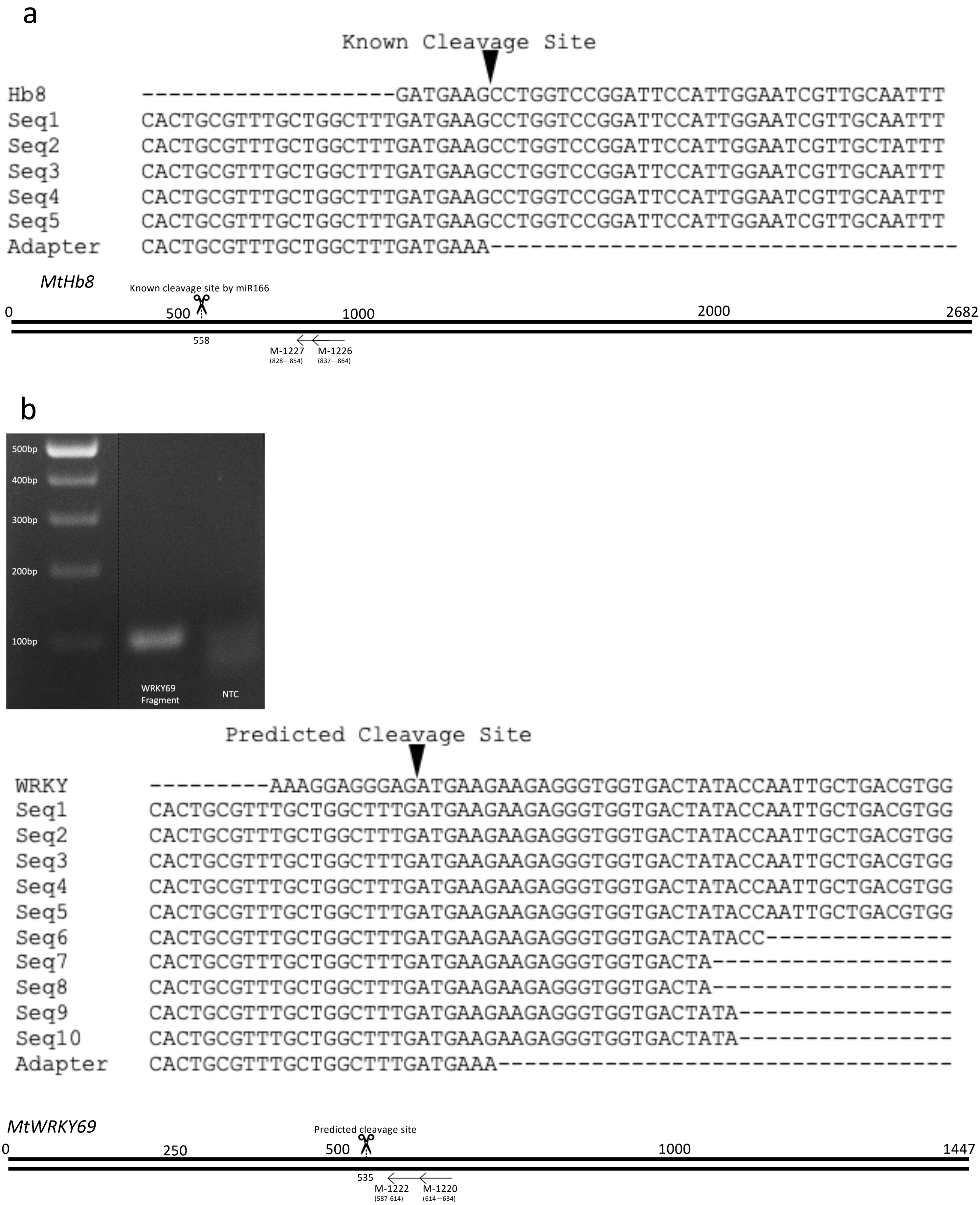

Fig. S4. 5' RLM RACE assay. a - (top) The alignment of the truncated fragment of *MtHb8* with the RLM RACE adapter and *MtHb8* itself. Each line of sequences represents a single colony. (bottom) *MtHb8* shown with the known cleavage site of *miR166* as well as the locations of the gene specific primers used. **b -** (top) Gel electrophoresis of 5' RLM RACE amplification targeting the *MtWRKY69* transcript using RNA from mycorrhizal roots. NTC: control. (middle) The alignment of the truncated fragment of *MtWRKY69* with the RLM RACE adapter and *MtWRKY69* itself. (bottom) *MtWRKY69* shown with predicted cleavage site as well as the locations of the gene specific primers used.

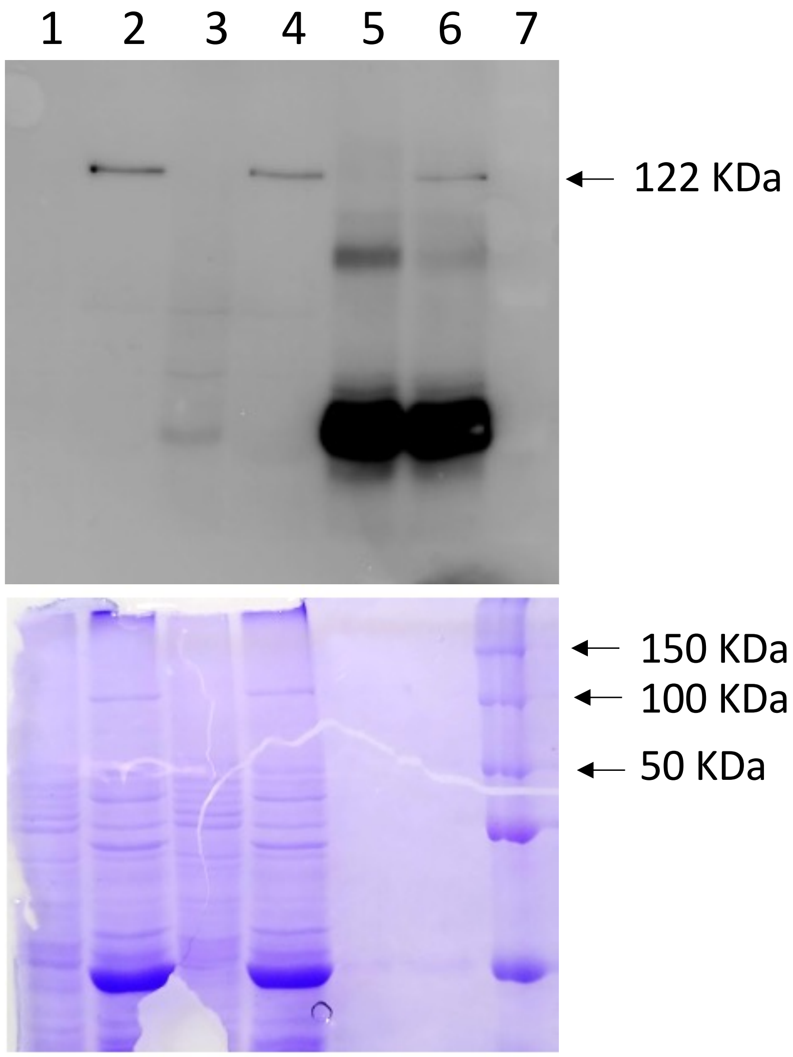

Fig. S5. Western blot using the anti-AGO1 antibody on proteins extracted from *M. truncatula* mycorrhizal samples. 1. root crude extract, 2. shoot crude extract, 3. root supernatant, 4. shoot supernatant, 5. root immunoprecipitated fraction, 6. shoot immunoprecipitated fraction, 7. molecular weight. Strong bands in 5 and 6 are the heavy and light chains of the antibodies used for IP.

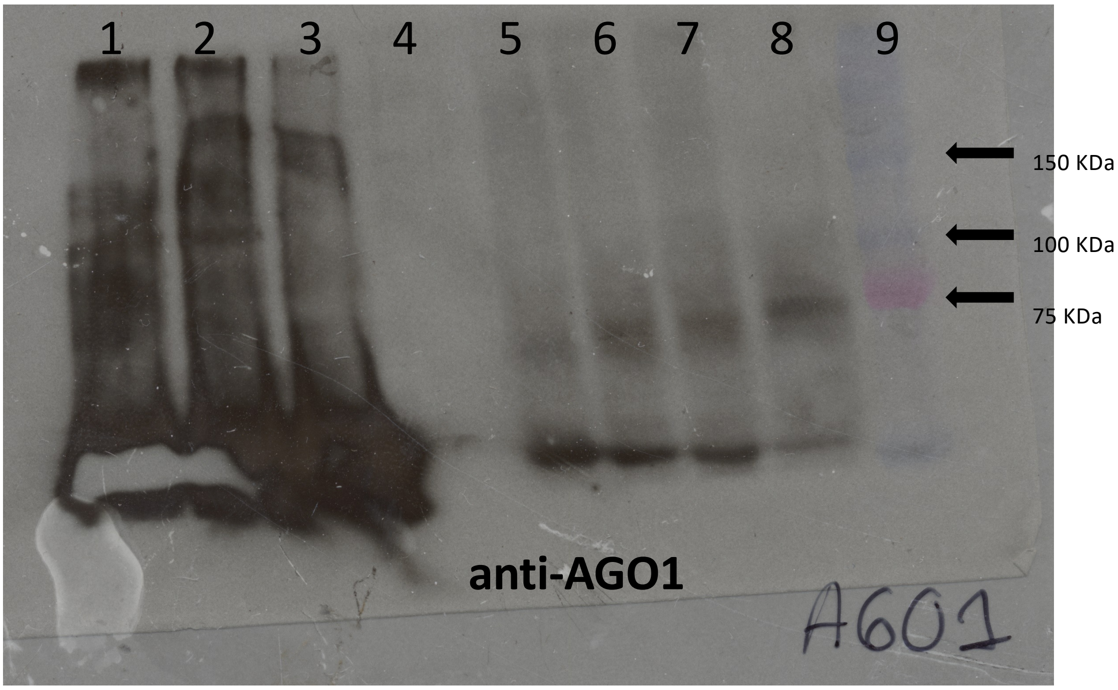

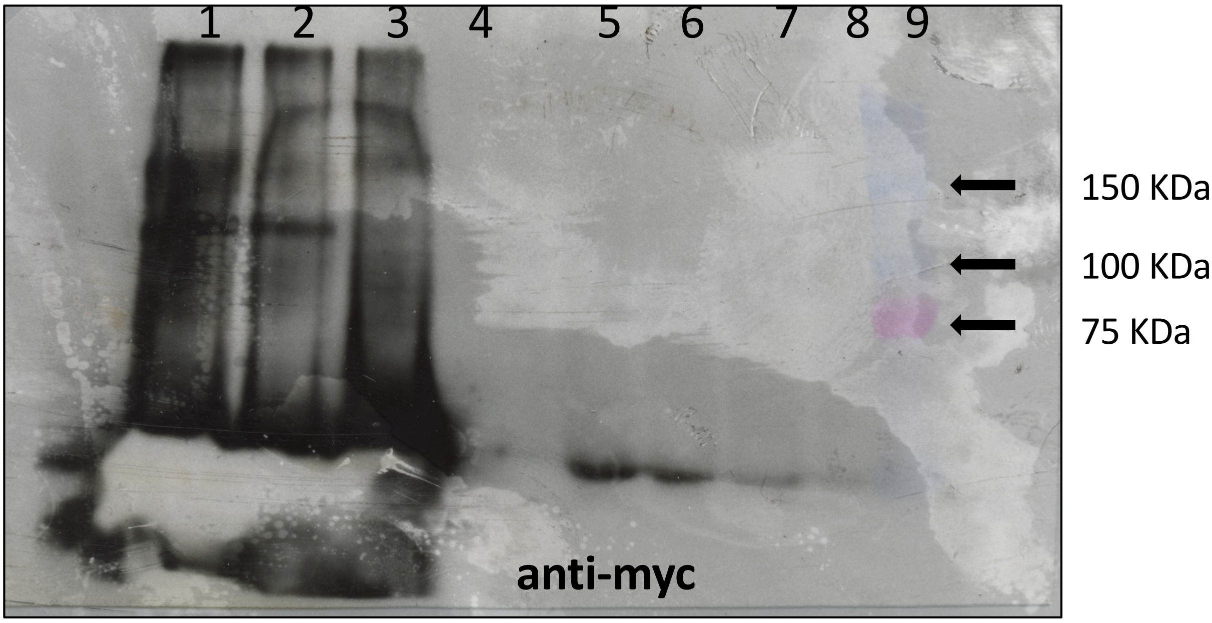

Fig. S6. Entire blots from figure 2d with anti-AGO1 (top) and anti-myc (bottom) antibodies. 1. Immunoprecipitated fraction using a combination of anti-AGO1 and anti-myc, 2. Immunoprecipitated fraction with anti-AGO1, 3. immunoprecipitated fraction with anti-myc, 4. control no antibody immunoprecipitated fraction, 5. crude extract corresponding to 1, 6. crude extract corresponding to 2, 7. crude extract corresponding to 3, 8. crude extract corresponding to 4, 9. ladder. Lanes 2, 3 and 4 are shown in Figure 2d.
